# Supplementary material for: The effect of a ketogenic diet and synergy with rapamycin in a mouse model of breast cancer
Source: PLoS One. 2020 Dec 3;15(12):e0233662. doi: 10.1371/journal.pone.0233662 (PMC7714189; doi:10.1371/journal.pone.0233662)
Supplement: S1 Table — (DOCX) [file pone.0233662.s001.docx]

**S1 Table**

Size and number of metastases in lungs from microscopic slide

| Diet | Diam. **largest** met(mm) | **Mean** Diam. largest met (mm) | **No. of mets** | **Mean no. of mets** |
| --- | --- | --- | --- | --- |
| st diet (sd) | 0.8 |  | \| 12 \| \| --- \| |  |
| **stdiet** | 1.75 | **1.52** | **18** | **18** |
| st diet | 2 |  | 24 |  |
| keto diet (kd) | 0.2 |  | 6 |  |
| **keto** | 1.5 | **0.90** | **32** | **15** |
| keto | 1 |  | 6 |  |
|  | **SD vs KD p=** | **0.10** |  | **NS** |
| St diet + low rapa | 4 |  | 4 |  |
| st diet + low rapa | 3 | **2.50** | **40** | **17** |
| st diet+ low rapa | 0.5 |  | 7 |  |
| keto+ low rapa | 1 |  | 50 |  |
| **Keto+ low rapa** |  | **0.83** | **0** | **19** |
| keto+ low rapa | 1.5 |  | 8 |  |
|  | **SD+R vs KD +R p=** | **0.70** |  | **NS** |
| SD + high rapa | 2 |  | 18 |  |
| **SD+ high rapa** | 1 | **1.67** | **16** | **17** |
| SD+ high rapa | 2 |  | 17 |  |
| keto+ high rapa | 0.8 |  | 12 |  |
| **keto + high rapa** | 1 | **0.93** | **8** | **23** |
| keto+ high rapa | 1 |  | 50 |  |
|  | **SD +R vs KD +R p=** | **0.19** |  | **NS** |
