# Supplementary material for: The effect of a ketogenic diet and synergy with rapamycin in a mouse model of breast cancer
Source: PLoS One. 2020 Dec 3;15(12):e0233662. doi: 10.1371/journal.pone.0233662 (PMC7714189; doi:10.1371/journal.pone.0233662)
Supplement: S1 File — (DOCX) [file pone.0233662.s002.docx]

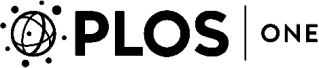


*PLOS ONE* Humane Endpoints Checklist

*PLOS ONE* manuscript number: _PONE-D-20-11420

*Complete the following if your study design includes death of a regulated animal as a likely outcome or planned experimental endpoint. Please also include all information in the Methods section of your manuscript.*

**ITEM 1.** **Describe whether humane endpoints* were used for all animals involved in the study.**

|  | Recommendation | Section/Paragraph |
| --- | --- | --- |
| *If humane endpoints* were used, report the following:* | |  |
| 1 | The specific criteria used to determine when animals should be euthanized | **Determination of moribund status and humane endpoints** line 131-137 |
| 2 | Once animals reached endpoint criteria, the amount of time elapsed before euthanasia | Same section, line 134-35 |
| 3 | Whether any animals died before meeting criteria for euthanasia | Same, line 135 |
| *If humane endpoints* were* ***not*** *used, report the following:* | |  |
| 1 | A scientific and ethical justification for the study design, including the reasons why humane endpoints could not be used, and discussion of alternatives that were considered but could not be used | Not applicable |
| 2 | Whether the institutional animal ethics committee specifically reviewed and approved the anticipated mortality in the study design | Not applicable. (Humane endpoints used.) |

**ITEM 2.** **Include the following details of the study design and outcomes.**

|  | Recommendation | Section/Paragraph |
| --- | --- | --- |
| 1 | The duration of the experiment | **Determination of humane endpoints** line131-137 |
| 2 | The numbers of animals used, euthanized, and found dead (if any); the cause of death for all animals | **Cancer model**; line 93-94; AND **Determination of endpoints**: line134-5. |
| 3 | How frequently animal health and behavior were monitored | **Determ. of Status**; line 131. |
| 4 | All animal welfare considerations taken, including efforts to minimize suffering and distress, use of analgesics or anaesthetics, or special housing conditions | **Determination of status & humane endpoints** line137-139  *AND*  **Housing and Husbandry** line 101-109 |
| 5 | Any special training in animal care or handling provided for research staff | **Special Training**  Line 109-111 |

Last modified December 2016


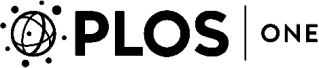


***Definition of a humane endpoint**

A humane endpoint is an experimental endpoint at which animals are euthanized when they display early markers associated with death or poor prognosis of quality of life, or specific signs of severe suffering or distress. Humane endpoints are used as an alternative to

allowing such conditions to continue or progress to death following the experimental intervention (“death as an endpoint”), or only euthanizing animals at the end of an

experiment. Before a study begins, researchers define the practical observations or measurements that will be used during the study to recognize a humane endpoint, based on anticipated clinical, physiological, and behavioral signs. These may include, for instance, body temperature or weight changes, tumor size or appearance, abnormal behaviors, pathological changes, ruffled fur, reduced mobility, body posture, or expression of specific body fluid markers. Please see the NC3Rs guidelines for more information.

**ARRIVE Guidelines**

*PLOS ONE* encourages authors to follow the [Animal Research: Reporting of In Vivo](http://www.nc3rs.org.uk/arrive-guidelines)

[Experiments (ARRIVE) guidelines](http://www.nc3rs.org.uk/arrive-guidelines) for all submissions describing laboratory-based animal research and to upload a completed [ARRIVE Guidelines Checklist](http://www.nc3rs.org.uk/sites/default/files/documents/Guidelines/NC3Rs%20ARRIVE%20Guidelines%20Checklist%20%28fillable%29.pdf) to be published as

supporting information.

Last modified December 2016 *PLOS ONE* Humane Endpoints Checklist 2
