# Supplementary material for: The effect of a ketogenic diet and synergy with rapamycin in a mouse model of breast cancer
Source: PLoS One. 2020 Dec 3;15(12):e0233662. doi: 10.1371/journal.pone.0233662 (PMC7714189; doi:10.1371/journal.pone.0233662)
Supplement: S2 File — (DOCX) [file pone.0233662.s003.docx]

| Bioanalytic Research Core assays of BHB 3 wks | | |
| --- | --- | --- |
| after Keto Mojo ( < 1sec after bleeding) | | |
| Groups | Robin | Keto Mojo |
|  | BHB | BHB mM |
| SD | 0.28 | 0.3 |
| SD | 0.40 | 0.4 |
| SD | 0.23 | 0.2 |
| SD | 0.54 | 0.5 |
| SD | 0.48 | 0.5 |
| SD | 0.54 | 0.6 |
| SD | 0.43 | 0.4 |
| SD | 0.17 | 0.2 |
| SD 0.4 | 0.20 | 0.2 |
| SD 0.4 | 0.31 | 0.2 |
| SD 0.4 | 0.28 | 0.3 |
| SD 4 | 0.16 | 0.2 |
| SD 4 | 0.20 | 0.2 |
| SD 4 | 0.39 | 0.2 |
| SD 4 | 0.30 | 0.3 |
| SD 4 | 0.26 | 0.3 |
| **Average** | **0.32** | **0.3** |
| StdDev | 0.13 | 0.1 |
| KD | 0.93 | 1.1 |
| KD | 1.00 | 1.2 |
| KD | 3.58 | 3.4 |
| KD | 4.35 | 4.5 |
| KD | 3.63 | 3.7 |
| KD | 2.55 | 2.7 |
| KD | 4.30 | 4.6 |
| KD | 2.46 | 2.7 |
| KD | 0.49 | 0.9 |
| KD 0.4 | 3.27 | 3.5 |
| KD 0.4 | 1.03 | 2.1 |
| KD 0.4 | 1.89 | 2.3 |
| KD 4 | 8.24 | 5.4 |
| KD 4 | 6.25 | 5.2 |
| KD 4 | 4.12 | 5.1 |
| KD 4 | 2.36 | 4.3 |
| **Average** | **3.15** | **3.3** |
| StdDev | 2.06 | 1.5 |
