# Supplementary material for: The effect of a ketogenic diet and synergy with rapamycin in a mouse model of breast cancer
Source: PLoS One. 2020 Dec 3;15(12):e0233662. doi: 10.1371/journal.pone.0233662 (PMC7714189; doi:10.1371/journal.pone.0233662)
Supplement: S3 File — (DOCX) [file pone.0233662.s004.docx]

| SD days |  |  |  |  |  |  |  |  |  |
| --- | --- | --- | --- | --- | --- | --- | --- | --- | --- |
| Gluc. values in mg/dl | Mouse 1 | Mouse 2 | Mouse 3 | Mouse 4 | Mouse 5 | Mouse 6 | Mouse 7 | Mouse 8 | Mouse 9 |
| 9-10 am | 187 | 312 | 213 | 188 | 169 | 264 | 176 | 284 | 232 |
| 1-2 pm | 246 | 198 | 298 | 161 | 264 | 211 | 273 | 231 | 186 |
| 5-6 pm | 221 | 171 | 197 | 173 | 179 | 167 | 220 | 178 | 159 |
| 6th day Average | 218 | 227 | 236 | 174 | 204 | 214 | 223 | 231 | 192 |
|  |  |  |  |  |  |  |  |  |  |
| 9-10 am | 222 | 264 | 205 | 201 | 176 | 243 | 175 | 261 | 224 |
| 1-2 pm | 189 | 199 | 273 | 181 | 244 | 197 | 254 | 194 | 181 |
| 5-6 pm | 176 | 171 | 194 | 165 | 174 | 172 | 219 | 169 | 161 |
| 12th | 196 | 211 | 224 | 182 | 198 | 204 | 216 | 208 | 189 |
|  |  |  |  |  |  |  |  |  |  |
| 9-10 am | 274 | 257 | 274 | 205 | 189 | 239 | 189 | 259 | 244 |
| 1-2 pm | 190 | 225 | 213 | 192 | 239 | 203 | 241 | 210 | 202 |
| 5-6 pm | 151 | 177 | 183 | 176 | 181 | 184 | 219 | 174 | 164 |
| 26th | 205 | 220 | 223 | 191 | 203 | 209 | 216 | 214 | 203 |
|  |  |  |  |  |  |  |  |  |  |
| 9-10 am | 237 | 247 | 214 | 204 | 231 | 240 | 209 | 249 | 229 |
| 1-2 pm | 196 | 215 | 259 | 187 | 194 | 213 | 224 | 223 | 191 |
| 5-6 pm | 171 | 185 | 167 | 164 | 180 | 179 | 189 | 168 | 166 |
| 35th | 201 | 216 | 213 | 185 | 202 | 211 | 207 | 213 | 195 |
|  |  |  |  |  |  |  |  |  |  |
| 9-10 am | 214 | 241 | 222 | 192 | 196 | 197 | 184 | 223 | 184 |
| 1-2 pm | 195 | 203 | 263 | 166 | 247 | 238 | 193 | 257 | 159 |
| 5-6 pm | 149 | 178 | 184 | 147 | 201 | 193 | 172 | 204 | 179 |
| 57th | 186 | 207 | 223 | 168 | 215 | 209 | 183 | 228 | 174 |
|  |  |  |  |  |  |  |  |  |  |
| KD days |  |  |  |  |  |  |  |  |  |
|  | 1 | 2 | 3 | 4 | 5 | 6 | 7 | 8 | 9 |
| 9-10 am | 187 | 164 | 287 | 185 | 214 | 192 | 276 | 235 | 161 |
| 1-2 pm | 224 | 173 | 234 | 246 | 203 | 256 | 251 | 264 | 229 |
| 5-6 pm | 209 | 224 | 202 | 239 | 209 | 204 | 184 | 188 | 186 |
| 6th | 207 | 187 | 241 | 223 | 209 | 217 | 237 | 229 | 192 |
|  |  |  |  |  |  |  |  |  |  |
| 9-10 am | 187 | 213 | 201 | 193 | 179 | 219 | 207 | 213 | 216 |
| 1-2 pm | 221 | 182 | 233 | 229 | 254 | 201 | 215 | 205 | 197 |
| 5-6 pm | 177 | 154 | 187 | 184 | 209 | 146 | 181 | 163 | 154 |
| 12th | 195 | 183 | 207 | 202 | 214 | 189 | 201 | 194 | 189 |
|  |  |  |  |  |  |  |  |  |  |
| 9-10 am | 141 | 139 | 168 | 213 | 197 | 189 | 218 | 184 | 175 |
| 1-2 pm | 198 | 162 | 223 | 189 | 164 | 194 | 181 | 171 | 191 |
| 5-6 pm | 167 | 139 | 185 | 143 | 152 | 166 | 159 | 173 | 165 |
| 26th | 169 | 147 | 192 | 182 | 171 | 183 | 186 | 176 | 177 |
|  |  |  |  |  |  |  |  |  |  |
| 9-10 am | 198 | 181 | 214 | 127 | 191 | 117 | 200 | 195 | 142 |
| 1-2 pm | 135 | 121 | 167 | 187 | 155 | 178 | 171 | 179 | 134 |
| 5-6 pm | 123 | 111 | 150 | 169 | 122 | 164 | 136 | 139 | 231 |
| 35th | 152 | 138 | 177 | 161 | 156 | 153 | 169 | 171 | 169 |
|  |  |  |  |  |  |  |  |  |  |
| 9-10 am | 121 | 84 | 106 | 181 | 143 | 93 | 116 | 89 | 181 |
| 1-2 pm | 115 | 117 | 142 | 143 | 110 | 96 | 164 | 191 | 133 |
| 5-6 pm | 102 | 111 | 115 | 117 | 89 | 168 | 134 | 154 | 109 |
| 57th | 113 | 104 | 121 | 147 | 114 | 119 | 138 | 145 | 141 |
|  |  |  |  |  |  |  |  |  |  |
| SD r0.4 days |  |  |  |  |  |  |  |  |  |
|  | 1 | 2 | 3 |  |  |  |  |  |  |
| 9-10 am | 279 | 215 | 245 |  |  |  |  |  |  |
| 1-2 pm | 233 | 268 | 247 |  |  |  |  |  |  |
| 5-6 pm | 214 | 210 | 222 |  |  |  |  |  |  |
| 6th | 242 | 231 | 238 |  |  |  |  |  |  |
|  |  |  |  |  |  |  |  |  |  |
| 9-10 am | 209 | 205 | 240 |  |  |  |  |  |  |
| 1-2 pm | 272 | 244 | 221 |  |  |  |  |  |  |
| 5-6 pm | 218 | 191 | 202 |  |  |  |  |  |  |
| 12th | 233 | 213 | 221 |  |  |  |  |  |  |
|  |  |  |  |  |  |  |  |  |  |
| 9-10 am | 207 | 216 | 251 |  |  |  |  |  |  |
| 1-2 pm | 283 | 253 | 229 |  |  |  |  |  |  |
| 5-6 pm | 223 | 235 | 206 |  |  |  |  |  |  |
| 26th | 238 | 235 | 229 |  |  |  |  |  |  |
|  |  |  |  |  |  |  |  |  |  |
| 9-10 am | 203 | 204 | 243 |  |  |  |  |  |  |
| 1-2 pm | 278 | 233 | 223 |  |  |  |  |  |  |
| 5-6 pm | 212 | 182 | 204 |  |  |  |  |  |  |
| 35th | 231 | 206 | 223 |  |  |  |  |  |  |
|  |  |  |  |  |  |  |  |  |  |
| 9-10 am | 235 | 281 | 301 |  |  |  |  |  |  |
| 1-2 pm | 304 | 241 | 236 |  |  |  |  |  |  |
| 5-6 pm | 261 | 222 | 230 |  |  |  |  |  |  |
| 57th | 267 | 248 | 256 |  |  |  |  |  |  |
|  |  |  |  |  |  |  |  |  |  |
| KD r0.4 days |  |  |  |  |  |  |  |  |  |
|  | 1 | 2 | 3 | 4 |  |  |  |  |  |
| 9-10 am | 208 | 216 | 179 | 173 |  |  |  |  |  |
| 1-2 pm | 259 | 191 | 247 | 235 |  |  |  |  |  |
| 5-6 pm | 212 | 184 | 207 | 186 |  |  |  |  |  |
| 6th | 226 | 197 | 211 | 198 |  |  |  |  |  |
|  |  |  |  |  |  |  |  |  |  |
| 9-10 am | 215 | 227 | 188 | 183 |  |  |  |  |  |
| 1-2 pm | 253 | 189 | 265 | 244 |  |  |  |  |  |
| 5-6 pm | 196 | 152 | 171 | 152 |  |  |  |  |  |
| 12th | 221 | 189 | 208 | 193 |  |  |  |  |  |
|  |  |  |  |  |  |  |  |  |  |
| 9-10 am | 175 | 132 | 149 | 189 |  |  |  |  |  |
| 1-2 pm | 201 | 133 | 190 | 143 |  |  |  |  |  |
| 5-6 pm | 184 | 168 | 174 | 121 |  |  |  |  |  |
| 26th | 187 | 144 | 171 | 151 |  |  |  |  |  |
|  |  |  |  |  |  |  |  |  |  |
| 9-10 am | 170 | 116 | 184 | 159 |  |  |  |  |  |
| 1-2 pm | 198 | 113 | 162 | 135 |  |  |  |  |  |
| 5-6 pm | 161 | 159 | 143 | 120 |  |  |  |  |  |
| 35th | 176 | 129 | 163 | 138 |  |  |  |  |  |
|  |  |  |  |  |  |  |  |  |  |
| 9-10 am | 127 | 89 | 149 | 117 |  |  |  |  |  |
| 1-2 pm | 156 | 119 | 134 | 89 |  |  |  |  |  |
| 5-6 pm | 145 | 83 | 113 | 79 |  |  |  |  |  |
| 57th | 143 | 97 | 132 | 95 |  |  |  |  |  |
|  |  |  |  |  |  |  |  |  |  |
| SD r4 days |  |  |  |  |  |  |  |  |  |
|  | 1 | 4 | 2 | 5 | 3 |  |  |  |  |
| 9-10 am | 214 | 216 | 265 | 306 | 197 |  |  |  |  |
| 1-2 pm | 276 | 259 | 233 | 273 | 193 |  |  |  |  |
| 5-6 pm | 237 | 219 | 215 | 207 | 240 |  |  |  |  |
| 6th | 242 | 231 | 238 | 262 | 210 |  |  |  |  |
|  |  |  |  |  |  |  |  |  |  |
| 9-10 am | 243 | 225 | 196 | 253 | 232 |  |  |  |  |
| 1-2 pm | 219 | 209 | 229 | 214 | 201 |  |  |  |  |
| 5-6 pm | 192 | 184 | 211 | 199 | 176 |  |  |  |  |
| 12th | 218 | 206 | 212 | 222 | 203 |  |  |  |  |
|  |  |  |  |  |  |  |  |  |  |
| 9-10 am | 223 | 252 | 198 | 206 | 262 |  |  |  |  |
| 1-2 pm | 255 | 214 | 192 | 268 | 216 |  |  |  |  |
| 5-6 pm | 230 | 185 | 273 | 213 | 179 |  |  |  |  |
| 26th | 236 | 217 | 221 | 229 | 219 |  |  |  |  |
|  |  |  |  |  |  |  |  |  |  |
| 9-10 am | 206 | 237 | 156 | 218 | 204 |  |  |  |  |
| 1-2 pm | 278 | 208 | 205 | 247 | 196 |  |  |  |  |
| 5-6 pm | 208 | 182 | 176 | 207 | 248 |  |  |  |  |
| 35th | 231 | 209 | 179 | 224 | 216 |  |  |  |  |
|  |  |  |  |  |  |  |  |  |  |
| 9-10 am | 217 | 243 | 242 | 209 | 205 |  |  |  |  |
| 1-2 pm | 278 | 221 | 208 | 256 | 265 |  |  |  |  |
| 5-6 pm | 231 | 193 | 183 | 237 | 223 |  |  |  |  |
| 57th | 242 | 219 | 211 | 234 | 231 |  |  |  |  |
|  |  |  |  |  |  |  |  |  |  |
| KD r4 days |  |  |  |  |  |  |  |  |  |
|  | 1 | 2 | 3 | 4 |  |  |  |  |  |
| 9-10 am | 247 | 193 | 200 | 232 |  |  |  |  |  |
| 1-2 pm | 221 | 224 | 235 | 209 |  |  |  |  |  |
| 5-6 pm | 206 | 206 | 219 | 201 |  |  |  |  |  |
| 6th | 225 | 208 | 218 | 214 |  |  |  |  |  |
|  |  |  |  |  |  |  |  |  |  |
| 9-10 am | 231 | 184 | 178 | 220 |  |  |  |  |  |
| 1-2 pm | 214 | 215 | 232 | 197 |  |  |  |  |  |
| 5-6 pm | 203 | 190 | 211 | 186 |  |  |  |  |  |
| 12th | 216 | 196 | 207 | 201 |  |  |  |  |  |
|  |  |  |  |  |  |  |  |  |  |
| 9-10 am | 208 | 163 | 162 | 187 |  |  |  |  |  |
| 1-2 pm | 191 | 195 | 217 | 173 |  |  |  |  |  |
| 5-6 pm | 167 | 155 | 146 | 144 |  |  |  |  |  |
| 26th | 189 | 171 | 175 | 168 |  |  |  |  |  |
|  |  |  |  |  |  |  |  |  |  |
| 9-10 am | 192 | 151 | 149 | 177 |  |  |  |  |  |
| 1-2 pm | 179 | 174 | 176 | 164 |  |  |  |  |  |
| 5-6 pm | 153 | 135 | 122 | 154 |  |  |  |  |  |
| 35th | 175 | 153 | 149 | 165 |  |  |  |  |  |
|  |  |  |  |  |  |  |  |  |  |
| 9-10 am | 178 | 143 | 133 | 167 |  |  |  |  |  |
| 1-2 pm | 183 | 164 | 175 | 172 |  |  |  |  |  |
| 5-6 pm | 142 | 139 | 148 | 150 |  |  |  |  |  |
| 57th | 168 | 149 | 152 | 163 |  |  |  |  |  |
