# Supplementary material for: The effect of a ketogenic diet and synergy with rapamycin in a mouse model of breast cancer
Source: PLoS One. 2020 Dec 3;15(12):e0233662. doi: 10.1371/journal.pone.0233662 (PMC7714189; doi:10.1371/journal.pone.0233662)
Supplement: Attachment — Submitted filename: Plos one review KD 6.2020.docx [file pone.0233662.s005.docx]

The manuscript entitled “The Effect of a Ketogenic Diet and Synergy with Rapamycin in a Mouse Model of Breast Cancer.” PONE-D-20-11420 The authors attempt to demonstrate that ketogenic diet combined with rapamycin inhibits breast cancer growth. There are both major and minor problems with this paper that make a thorough review difficult.

Some minor problems that need to be addressed: Line 73 of introduction states, “effects in humans” and then lists references that are feasibility studies, and do not show effectiveness. Researchers must be very careful with language when discussing human trials. Line 82 list the protein/carb/fat content but does not discuss the kind of fat used. We have found that the lipid/PUFA content of the KD is very important for tumor growth inhibition. Please list the complete content of the diet. Line 183 a decimal is missing. Figure 1B, the initial blood glucose is very high in all the animals. Even after 11 days the glucose level is very high in KD groups. This is very surprising and counterintuitive. Sometimes blood glucose in mice spikes if they are scared and the time of the blood draw and you don’t act very quickly. Therefore, hemoglobin A1C might be a better indicator of true glucose control. Figure 1D insulin spelling on the axis and no indication of statistics performed. Figure 2A how many mice were in each group. It should be in the legend. The way that you measure tumor growth hides important data. Did you count the number of tumors arising on each mouse (tells us if initiation is affected); vs tumor volume of a single tumor (growth inhibition).

Some major problems to be addressed: line 108 indicates that there was only one investigator measure the mice. How could you keep that person blinded? Tumor size measurements has been shown to be subject to UNintentional biasing. It is essential that the investigator be blinded. Also, the general health of the mice needs to be investigated. Were they lethargic? Was there a change in their coat? Is there any other indication that the mice we made ill by the treatment? Lastly, the lung needs to be examined by a blinded pathologist to determine the number and stage of the nodules.

In general, there might be interesting information within the text but because the data is not adequately described it is impossible for me to adequately review it.
