# Supplementary material for: The effect of a ketogenic diet and synergy with rapamycin in a mouse model of breast cancer
Source: PLoS One. 2020 Dec 3;15(12):e0233662. doi: 10.1371/journal.pone.0233662 (PMC7714189; doi:10.1371/journal.pone.0233662)
Supplement: Attachment — Submitted filename: Plos one re-review KD 8.2020.docx [file pone.0233662.s006.docx]

The manuscript entitled “The Effect of a Ketogenic Diet and Synergy with Rapamycin in a Mouse Model of Breast Cancer.” PONE-D-20-11420 The authors attempt to demonstrate that ketogenic diet combined with rapamycin inhibits breast cancer growth. There are both major and minor problems with this paper that make a thorough review difficult.

Some minor problems that need to be addressed: Line 73 of introduction states, “effects in humans” and then lists references that are feasibility studies, and do not show effectiveness. Researchers must be very careful with language when discussing human trials.

As described in answer to Reviewer #1 we have changed the references.

I do not think you have adequately addressed my concerns. The first sentence and line 74 still imply effectiveness of KD in humans. None of the references 1-4 or 15-17 support effectiveness in human studies. There are numerous small studies that show feasibility of KD only. Reference 6 (probably changed for reference 5?) is a small pilot study that concludes nothing about prolonged survival. In addition, all references need to be double checked for accuracy.

Line 82 list the protein/carb/fat content but does not discuss the kind of fat used. We have found that the lipid/PUFA content of the KD is very important for tumor growth inhibition. Please list the complete content of the diet.

The diet composition is now listed. Both diets contained fat derived from cocoa butter, obviously with the same ratio of saturated, monounsaturated and polyunsaturated fats; of course, the total amount of fat was very different. It is certainly plausible that other ratios of PUFA to saturated fats would have effects beyond those we’ve shown. It would be interesting and important to understand those effects, but that is a different study than we sought to accomplish.

Thank you for the though listing of fat/PUFA content. This is important data for other researchers wishing to build upon when answering the specific types of nutrients to include in a diet.

Line 183 a decimal is missing.

Figure 1B, the initial blood glucose is very high in all the animals. Even after 11 days the glucose level is very high in KD groups. This is very surprising and counterintuitive. Sometimes blood glucose in mice spikes if they are scared and the time of the blood draw and you don’t act very quickly. Therefore, hemoglobin A1C might be a better indicator of true glucose control.

This is a good point and interesting observation. This is not a common breed of mouse and the high glucose may be related to the physiology but, at this point, it is a reproducible measurement. It has also been our observation in other mouse and rat experiments that high and irregular glucose concentrations can be observed. Despite the high glucose value, it was interesting, and we believe important, that insulin remained low in the ketogenic diet group, and not in the standard diet group. It appears that in at least some rodent breeds glucose levels are not regulated nearly as tightly as in humans. We did not measure glucagon, and perhaps that would have some bearing.

# Glucose and insulin level data if very important especially because this study might be translated into humans. It is important to discuss the limitations of model system. The original strain of mice in which this model system was derived, B6 have very high fasting and non-fasting glucose. They also have abnormal insulin responses. These mice might resemble diabetic or glucose intolerant humans more so than healthy humans. A good reference for this discussion is: **Glucose Homeostasis and Tissue Transcript Content of Insulin Signaling Intermediates in Four Inbred Strains of Mice: C57BL/6, C57BLKS/6, DBA/2, and 129X1** *Endocrinology*, Volume 145, Issue 7, 1 July 2004. In Figure 1 the KD returns the glucose level down to what would be in wildtype strains or healthy humans not on a KD. For clarity please list the strain of mouse used in the abstract; add comparisons of glucose and insulin levels of wild type mice in the results; and the limitations to translation of the model system to humans in the discussion. Another important point for the discussion is that the diet in this study is very low in carbohydrates and does not resemble most human KDs.

Figure 1D insulin spelling on the axis and no indication of statistics performed. Figure 2A how many mice were in each group.

It should be in the legend.

The number of mice in each group is not in the figure legend. The abstract should read 3-9 mice per group on line 20. Line 194 still incorrectly says 6 groups of 34 mice each. It is upsetting to this reviewer that the problem was not adequately addressed the first time. It looks like only 7 mice are in the survival graph in the SD.

The way that you measure tumor growth hides important data. Did you count the number of tumors arising on each mouse (tells us if initiation is affected); vs tumor volume of a single tumor (growth inhibition).

**: “Because there are many tumors of different sizes, an ideal presentation is not obvious. It would surely be interesting to understand initiation better, but this was not the point of the study.

Fine, but if you have the lungs you could report the number of nodules.

Some major problems to be addressed: line 108 indicates that there was only one investigator measure the mice. How could you keep that person blinded? Tumor size measurements has been shown to be subject to UNintentional biasing. It is essential that the investigator be blinded. Also, the general health of the mice needs to be investigated.

Tumor size was measured with calipers. We do not believe bias was involved but we must acknowledge that it’s possible to harbor an inadvertent bias, ie to ‘wish’ for a particular result. So we thank the reviewer for pointing out this possibility. However, even if bias of any kind was involved at the time of caliper measurements, lung weight (vastly tumor) and body were weighed at post-mortem. There is no personal judgement involved in weight measurements. If bias was involved at the point of animal euthanasia (i.e. if tumors were measured as too large in order to inadvertently delay the euthanasia) then the weight measurement of metastases to the lungs should not have turned out lower than in the standard diet group. The above said, we appreciate the insight. Whereas we did not even consider the idea of blinding the caliper measurements in the present study, we will certainly do so in future studies. I have double checked with the IACUC who have informed me that this is indeed the standard.

Thank you for your considered response. I agree that the large difference in the tumor volume was unlikely due to unintentional bias. I also think that lung weight is valid measurement to report. The line about an individual measurer should be left in the manuscript so the limitations of the study are clearly understood.

Were they lethargic? Was there a change in their coat? Is there any other indication that the mice we made ill by the treatment?

The general health of the mice was indeed evaluated daily, as in the text. In particular, the ability of the mice to reach their food or water (or lethargy, i.e. the inability to reach the food or water) was assessed regularly, as indicated. The coat was evaluated weekly, but no changes were observed. Since inability to reach food was the justification for euthanasia, as determined by our IACUC, we did not indicate animal coat quality in the text; a clear coat did not change our decision. The animals were dragging due to a huge burden of primary and especially metastatic disease, as their lung to total body weight ratios in our table indicate. The coat quality, which may otherwise be important, in this instance had no role in determining the endpoint of this study.

I understand that the condition of the mice regarding tumor burden was evaluated but what about diet and rapamycin. Did you see a change in the mice before the tumor endpoint that might indicate that they did not tolerate the diet or drug?

Lastly, the lung needs to be examined by a blinded pathologist to determine the number and stage of the nodules.

The lungs were indeed evaluated by a blinded pathologist (co-author Dr. Fineberg), as indicated in the text. But the most fundamental data, from the perspective of tumor metastatic mass, was the measurement of lung weight. A pathologic figure was provided, but only as representative of the lung data, as explicitly stated in the legend. Actual counting of mets and sizes of mets by the pathologist were statistically far less meaningful than overall metastatic lung mass and weight. The overall tumor mass provides the integral of tumor mass from what would have to be innumerable slides. The measure of total metastatic mass to determine overall metastatic information was approved by our pathology co-author who read and reviewed the manuscript, as indicated in our submission

Line 172 should say blinded pathologist. For figure 4, the number of mets in the lung is interesting and important and should be included especially considering the experimental limitations on the other data. If the data is good enough to be included as a qualitative figure than it should be quantified.

There are still multiple minor issues including but not comprehensively; line 194 says 4 week old mice started the diet while line 237 says 6 week old. It is never explained if the mice go off both the treatment and the diet after 5 weeks or do they continue on the diet until death? Too many period on line 201. Line 88 has grams 3 different ways. More than one font is used.
