# Supplementary material for: The effect of a ketogenic diet and synergy with rapamycin in a mouse model of breast cancer
Source: PLoS One. 2020 Dec 3;15(12):e0233662. doi: 10.1371/journal.pone.0233662 (PMC7714189; doi:10.1371/journal.pone.0233662)
Supplement: Attachment — Submitted filename: Response to reviewers 9-30-2020.docx [file pone.0233662.s007.docx]

The manuscript entitled “The Effect of a Ketogenic Diet and Synergy with Rapamycin in a Mouse Model of Breast Cancer.” PONE-D-20-11420 The authors attempt to demonstrate that ketogenic diet combined with rapamycin inhibits breast cancer growth. There are both major and minor problems with this paper that make a thorough review difficult.

Some minor problems that need to be addressed: Line 73 of introduction states, “effects in humans” and then lists references that are feasibility studies, and do not show effectiveness. Researchers must be very careful with language when discussing human trials.

As described in answer to Reviewer #1 we have changed the references.

I do not think you have adequately addressed my concerns. The first sentence and line 74 still imply effectiveness of KD in humans. None of the references 1-4 or 15-17 support effectiveness in human studies. There are numerous small studies that show feasibility of KD only. Reference 6 (probably changed for reference 5?) is a small pilot study that concludes nothing about prolonged survival. In addition, all references need to be double checked for accuracy.

Reference 5 and reference 6 do indeed need to be swapped. We have done this.

We owe Dr. Klement an apology for improperly describing data that he illustrated very well in his review (ref. 37), now properly included. This review as well as some of the other articles now cited were not available on-line in our institution’s library at the time we wrote our response (the library as well as the institution were having IT difficulties, partly due to COVID19 and staffing) but these references have since become available. We were in the unfortunate position of quoting articles that Dr. Klement recommended without the opportunity to read them properly.

In this comment I agree with the reviewer in many respects. We didn’t actually use the term effectiveness. The terms “effects” and “efficacy” are employed in several sentences, but not in reference to the articles Dr. Klement describes. The first sentence and line 74 both use the word “effects”, but in the context of animal studies, not humans. There is also a distinction between efficacy from effectiveness when used in a medical context. Effectiveness is an overall measure used when patients take drugs clinically, including an overall reduction of benefit when patients stop taking drugs due to side effects, for example. The term efficacy is limited to *controlled trials*, where behavior of the subjects is controlled, by definition, and where efficacy is measured among those who successfully take the medication.

In any event our study’s goal was to reduce side effects so that an increase in the overall *effectiveness* of combined therapies might be realized, perhaps eventually in humans. So, perhaps ironically, we should actually have used the term effectiveness, not efficacy, with reference to our hoped for future objective in patients. We have swapped in that term in the relevant sentences of the Introduction as well as in the Discussion.

Given these distinctions, while we agree with the reviewer that phase 1 trials are designed to measure feasibility and safety and, *are not designed* to measure efficacy or effectiveness, it is not at all unusual for such measures to be reported anyway. For illustrative purposes, in refs 15-17, itemized by Dr. Klement, the trials were designed to measure safety and feasibility, but various outcome effects (overall survival, for example) were indeed measured and reported.

I hope the above clarifies what we meant to say.

Line 82 list the protein/carb/fat content but does not discuss the kind of fat used. We have found that the lipid/PUFA content of the KD is very important for tumor growth inhibition. Please list the complete content of the diet.

The diet composition is now listed. Both diets contained fat derived from cocoa butter, obviously with the same ratio of saturated, monounsaturated and polyunsaturated fats; of course, the total amount of fat was very different. It is certainly plausible that other ratios of PUFA to saturated fats would have effects beyond those we’ve shown. It would be interesting and important to understand those effects, but that is a different study than we sought to accomplish.

Thank you for the though listing of fat/PUFA content. This is important data for other researchers wishing to build upon when answering the specific types of nutrients to include in a diet.

Line 183 a decimal is missing.

I did find a missing decimal on line 219 in the revised version and corrected this. Thank you. (I have line 183 in front of me and see no missing decimals. The number on line 183 is a statistical significance value of 0.05. This could be an issue of changing line numbers during editing.)

Figure 1B, the initial blood glucose is very high in all the animals. Even after 11 days the glucose level is very high in KD groups. This is very surprising and counterintuitive. Sometimes blood glucose in mice spikes if they are scared and the time of the blood draw and you don’t act very quickly. Therefore, hemoglobin A1C might be a better indicator of true glucose control.

This is a good point and interesting observation. This is not a common breed of mouse and the high glucose may be related to the physiology but, at this point, it is a reproducible measurement. It has also been our observation in other mouse and rat experiments that high and irregular glucose concentrations can be observed. Despite the high glucose value, it was interesting, and we believe important, that insulin remained low in the ketogenic diet group, and not in the standard diet group. It appears that in at least some rodent breeds glucose levels are not regulated nearly as tightly as in humans. We did not measure glucagon, and perhaps that would have some bearing.

# Glucose and insulin level data if very important especially because this study might be translated into humans. It is important to discuss the limitations of model system. The original strain of mice in which this model system was derived, B6 have very high fasting and non-fasting glucose. They also have abnormal insulin responses. These mice might resemble diabetic or glucose intolerant humans more so than healthy humans. A good reference for this discussion is: **Glucose Homeostasis and Tissue Transcript Content of Insulin Signaling Intermediates in Four Inbred Strains of Mice: C57BL/6, C57BLKS/6, DBA/2, and 129X1** *Endocrinology*, Volume 145, Issue 7, 1 July 2004. In Figure 1 the KD returns the glucose level down to what would be in wildtype strains or healthy humans not on a KD. For clarity please list the strain of mouse used in the abstract; add comparisons of glucose and insulin levels of wild type mice in the results; and the limitations to translation of the model system to humans in the discussion. Another important point for the discussion is that the diet in this study is very low in carbohydrates and does not resemble most human KDs.

We thank the reviewer for pointing out our omission. We have surely added that effects seen in mouse models cannot be assumed to apply to humans. However, if anything, a higher glucose level in a mouse vs. a human (on a KD) would argue for a better effect in the human than the mouse. Humans also can reach higher levels of ketosis than mice, even though they consume less restrictive ketogenic diets, so the effects in mice again may argue for an even better response in humans. Nonetheless, we agree that we cannot assume translation to the human without further study and we have indicated this.

We have observed wide variations in blood glucose responses in mice in previous studies of our own. We have chosen to not discuss these aspects of mouse physiologic behavior in detail. This is a complex subject and has no direct bearing on the primary results showing prolonged survival. We’ve pointed out that rapamycin itself causes hyperglycemia in humans, although not in mice in doses such as those administered. The observation that hyperglycemia without hyperinsulinemia was seen initially on the ketogenic diet in this mouse model and then reverted to normal glucose concentrations during the ketogenic diet is surely an unusual finding. The overall pattern however shows distinct differences between a continued KD vs. a continued SD, where in the latter neither the high glucose levels (nor the insulin levels) correct over time. The suggestion that an initial anxiety reaction (provoking epinephrine secretion with attendant hyperglycemia, for example) is quite plausible, but we did not obtain epinephrine levels.

Further discussion on the potential hormonal interactions would indeed be interesting but would nonetheless be largely speculative without additional data. At this point the speculation involved would be fodder for further criticism but would contribute little to our primary observations or the reasons for our study. This kind of discussion would be more appropriate to a comprehensive review article of hyperglycemia in mice and the variations seen in different mouse breeds.

In summary, the desired and expected physiologic effect of ketosis measured in our mouse model was, despite the extreme level of carbohydrate restriction, if anything, less pronounced than an expected effect in humans. We elicited 2 mM ketone body concentrations in mouse serum, whereas a lesser extent of CHO restriction normally achievable by humans can produce 4-5 mM or more. These differences between mouse and human models have no obvious bearing on our hypothesis or on our primary observations. Similar to the complex issue of glycemic responses in diverse mouse species above, this discussion would be more appropriate to a review article.

Figure 1D insulin spelling on the axis and no indication of statistics performed. Figure 2A how many mice were in each group.

It should be in the legend.

The number of mice in each group is not in the figure legend. The abstract should read 3-9 mice per group on line 20. Line 194 still incorrectly says 6 groups of 34 mice each. It is upsetting to this reviewer that the problem was not adequately addressed the first time. It looks like only 7 mice are in the survival graph in the SD.

We apologize for not recognizing these errors, presumably resulting from the mental blur of reviewing the same text too many times. We’ve corrected the numbers in the paper as well as added the recommended numbers to the abstract. The numbers of mice in the SD at start are 9, as indicated.

The way that you measure tumor growth hides important data. Did you count the number of tumors arising on each mouse (tells us if initiation is affected); vs tumor volume of a single tumor (growth inhibition).

**: “Because there are many tumors of different sizes, an ideal presentation is not obvious. It would surely be interesting to understand initiation better, but this was not the point of the study.

Fine, but if you have the lungs you could report the number of nodules.

Thank you for your comment. We have included data from a pathologic slide that was evaluated. It is in our supplemental data.

Tumor initiation is indeed interesting, and it is represented in our model by the spontaneous tumor initiation in breast tissue. Metastases do not reflect initiation as they arise due to additional mutations which permit breaking off from the primary tumor, lodging and growing in distant tissues, etc. The lung metastases were innumerable and these numbers were not reported by the pathologist. Larger nodule sizes were reported in the supplemental data, but these data were limited and differences between groups were not statistically significant, as indicated in the text. As you can see from the representative slide, many were microscopic. The bulk of the paraffin block was not used for staining. It was saved for RNA sequencing analysis and proteomics, both sent to other laboratories. These analyses were delayed by COVID and could not be included in this paper. At this point we don’t know if these samples will ever be analyzed or even recovered.

Some major problems to be addressed: line 108 indicates that there was only one investigator measure the mice. How could you keep that person blinded? Tumor size measurements has been shown to be subject to UNintentional biasing. It is essential that the investigator be blinded. Also, the general health of the mice needs to be investigated.

Tumor size was measured with calipers. We do not believe bias was involved but we must acknowledge that it’s possible to harbor an inadvertent bias, ie to ‘wish’ for a particular result. So we thank the reviewer for pointing out this possibility. However, even if bias of any kind was involved at the time of caliper measurements, lung weight (vastly tumor) and body were weighed at post-mortem. There is no personal judgement involved in weight measurements. If bias was involved at the point of animal euthanasia (i.e. if tumors were measured as too large in order to inadvertently delay the euthanasia) then the weight measurement of metastases to the lungs should not have turned out lower than in the standard diet group. The above said, we appreciate the insight. Whereas we did not even consider the idea of blinding the caliper measurements in the present study, we will certainly do so in future studies. I have double checked with the IACUC who have informed me that this is indeed the standard.

Thank you for your considered response. I agree that the large difference in the tumor volume was unlikely due to unintentional bias. I also think that lung weight is valid measurement to report. The line about an individual measurer should be left in the manuscript so the limitations of the study are clearly understood.

Thank you for your remark as well. We have indicated the individual measurer.

Were they lethargic? Was there a change in their coat? Is there any other indication that the mice we made ill by the treatment?

The general health of the mice was indeed evaluated daily, as in the text. In particular, the ability of the mice to reach their food or water (or lethargy, i.e. the inability to reach the food or water) was assessed regularly, as indicated. The coat was evaluated weekly, but no changes were observed. Since inability to reach food was the justification for euthanasia, as determined by our IACUC, we did not indicate animal coat quality in the text; a clear coat did not change our decision. The animals were dragging due to a huge burden of primary and especially metastatic disease, as their lung to total body weight ratios in our table indicate. The coat quality, which may otherwise be important, in this instance had no role in determining the endpoint of this study.

I understand that the condition of the mice regarding tumor burden was evaluated but what about diet and rapamycin. Did you see a change in the mice before the tumor endpoint that might indicate that they did not tolerate the diet or drug?

We’ve indicated all the changes that were observed. None of the already described changes could be attributed to rapamycin, and none suggested intolerance to either diet or to the drug. As mentioned, there were changes neither in the coat quality nor were there behavioral changes other than those already mentioned. We saw no intolerance to rapamycin or diet and saw no reason to raise these issues, particularly while mice have long been studied with ketogenic diets as well as with rapamycin at moderate doses without such intolerance reported. (Ketogenic diets administered by Tisdale et al, as in our reference go back to the 1980’s. Rapamycin has been used in mice studies for years, and just recently reported in Nature, (our ref. 8) without such effects noted.)

Lastly, the lung needs to be examined by a blinded pathologist to determine the number and stage of the nodules.

The lungs were indeed evaluated by a blinded pathologist (co-author Dr. Fineberg), as indicated in the text. But the most fundamental data, from the perspective of tumor metastatic mass, was the measurement of lung weight. A pathologic figure was provided, but only as representative of the lung data, as explicitly stated in the legend. Actual counting of mets and sizes of mets by the pathologist were statistically far less meaningful than overall metastatic lung mass and weight. The overall tumor mass provides the integral of tumor mass from what would have to be innumerable slides. The measure of total metastatic mass to determine overall metastatic information was approved by our pathology co-author who read and reviewed the manuscript, as indicated in our submission.

Line 172 should say blinded pathologist. For figure 4, the number of mets in the lung is interesting and important and should be included especially considering the experimental limitations on the other data. If the data is good enough to be included as a qualitative figure than it should be quantified.

The number of overall mets was innumerable but was quantified per high power field by the pathologist based on the slide she evaluated. It is not reasonable to extrapolate macroscopic numbers from one or several microscopic slides. We have discussed this with our pathologist. The reason we reported the weight of the lung metastases was precisely because an attempt at enumeration of metastases from slides had serious statistical limitations. We indicated this in the text. We have included the pathologic enumeration of metastases from slides as provided to us by our blinded pathologist in the Supplemental Data.

There are still multiple minor issues including but not comprehensively; line 194 says 4 week old mice started the diet while line 237 says 6 week old. It is never explained if the mice go off both the treatment and the diet after 5 weeks or do they continue on the diet until death? Too many period on line 201. Line 88 has grams 3 different ways. More than one font is used.

Thank you for noticing these inconsistencies. The mice were received at approximately 4 weeks of age from Jackson Labs and were started on their diets at approximately 6 weeks, on which they remained until death by euthanasia. This information has been clarified in the text. Multiple “gram” spellings have been condensed to ‘gm’ in all cases. I can’t find extra periods on line 201 or anywhere nearby. My apologies, as I’ve now been over that vicinity many times. I have also double checked the font and it remains Arial 12 throughout the paragraphs, with the exception of the Volume equation (in the Tumor and Survival Measurement section) which was most clearly portrayed using Cambria Math font.
