# Supplementary material for: The effect of a ketogenic diet and synergy with rapamycin in a mouse model of breast cancer
Source: PLoS One. 2020 Dec 3;15(12):e0233662. doi: 10.1371/journal.pone.0233662 (PMC7714189; doi:10.1371/journal.pone.0233662)
Supplement: Attachment — Submitted filename: Response to Reviewers v. 4 10-21-2020.docx [file pone.0233662.s008.docx]

Reviewer #1: My previous comment appears to NOT have been taken into account; I accept the paper but would like the authors to change reference 37 into Klement et al. 2020, Med Oncol 37:14 (https://pubmed.ncbi.nlm.nih.gov/31927631/).

Response: Our apologies. We have changed the reference as requested.

Reviewer #2: I do not feel that the authors adequately addressed my concerns from prior reviews. Importantly, my concerns with discussing the limitations of the murine model regarding translation to humans. The murine mouse model they have used is based on a diabetic mouse and this is important for the translation and deserved mention in the discussion. They have added reference 37, which is a review paper that only cites murine model data and they have written that it is a human trial. In both previous reviews I asked them to be cautious about overstating human trial results. Instead of heeding my advice they lectured me on the difference of effectiveness and efficacy. The added pathology quantification data is important but underrepresented by putting it in supplemental.

Response: We have changed reference 37 as also requested by Reviewer #1. The new review indeed addresses human studies.

We also have added the reference recommended by Reviewer #2 regarding transgenic mice, particularly the insulin resistance of our mouse strain’s genetic background. Despite our initial skepticism about the added value of this information, it is indeed relevant, and we have added to the discussion accordingly.

We disagree that the pathology quantification data adds to the fundamental information required in the paper. The numbers of tumors and the size information is too sparse to be statistically significant. The statistically significant data is already within the fundamental figures included. These data are based on macroscopic weight measurements, not a single microscopic slide. But if the editors wish to move this slide into an additional fundamental figure for the paper, we will accept their judgment.
